# That sinking feeling: Suspended sediments can prevent the ascent of coral egg bundles

**DOI:** 10.1038/srep21567

**Published:** 2016-02-22

**Authors:** Gerard F. Ricardo, Ross J. Jones, Andrew P. Negri, Roman Stocker

**Affiliations:** 1 Australian Institute of Marine Science, Townsville, 4810, Queensland, and Perth, 6009, Western Australia, Australia; 2 Centre for Microscopy, Characterisation and Analysis, The University of Western Australia, Perth, 6009, Western Australia, Australia; 3 Western Australian Marine Science Institution, Perth, 6009, Western Australia, Australia; 4 Oceans Institute, University of Western Australia, Perth, 6009, Western Australia, Australia; 5 Department of Civil, Environmental and Geomatic Engineering, ETH Zurich, 8093 Zurich, Switzerland

## Abstract

Spawning synchrony represents a common reproductive strategy in sessile marine organisms and for broadcast spawning corals, buoyancy of egg-sperm bundles is critical to maximise fertilisation at the ocean surface. Here we demonstrate a novel threat to coral reproduction whereby buoyant egg-sperm bundles intercept and are “ballasted” by sediment grains on their journey to the ocean surface, preventing them from reaching the ocean surface and greatly reducing egg-sperm encounter rates. Empirical observations of this mechanism are successfully captured by a mathematical model that predicts the reduction in ascent probability and egg-sperm encounters as a function of sediment load. When applied to 15 m deep reefs, the model predicts that 10% and 50% reductions in egg-sperm encounters occur at 35 mg L^−1^ and 87 mg L^−1^ suspended sediment concentrations, respectively, and for a 5 m deep reef a 10% reduction occurs at 106 mg L^−1^. These concentrations are commonly associated with sediment plumes from dredging or natural resuspension events. The potential for sediments to sink coral gametes highlights the need to carefully manage the timing of turbidity-generating human activities near reefs during spawning periods.

Declining water quality is a major threat to coral reefs. Natural resuspension events^1^, river runoff^2^ and sediment plumes associated with human activities including dredging operations^3^,^4^ elevate the concentrations of suspended solids (SS) in marine waters. Elevated SS can attenuate light availability required for primary production, reduce feeding efficiency in filter feeders, and settle onto sessile invertebrates such as corals, reducing solute exchange and causing partial or complete mortality^5^. Elevated SS can also negatively impact the early life history stages of coral including fertilisation, larval development, settlement and post-settlement survival^6^,^7^. However, the vulnerability to suspended solids of reproductive stages prior to fertilisation has not been considered^8^.

The coordinated release of coral gametes, packaged as buoyant egg-sperm bundles within a mucous sheath, is the culmination of months of gametogenic synchronization in broadcast spawning species^9^. The ascent through the water column, timely arrival at the surface, and release of gametes from the bundles are critical for increasing egg-sperm encounter rates, and subsequent fertilisation. We hypothesized that, during ascent, the bundle can intercept suspended sediment grains that stick to its mucous coating. Here we demonstrate this mechanism through experimental observations and mathematical modelling and show that the ballasting effect of intercepted sediments is often sufficient to reverse the ascent, causing a sizeable fraction of bundles to sink. The detrimental impact of this loss of bundles reaching the water surface on egg-sperm encounters is nonlinear because the bundles carry both eggs and sperm, and the encounter rate is proportional to the product of their respective concentrations at the surface. Even for bundles that remain positively buoyant, reaching the surface might be delayed, further reducing egg-sperm encounter rates. This is the first study to examine the effects of environmental pressures on the success of gamete ascent, a critical step in recruitment success, which serves to replenish and facilitate recovery of coral reefs^10^.

## Results and Discussion

**Figure 1:**
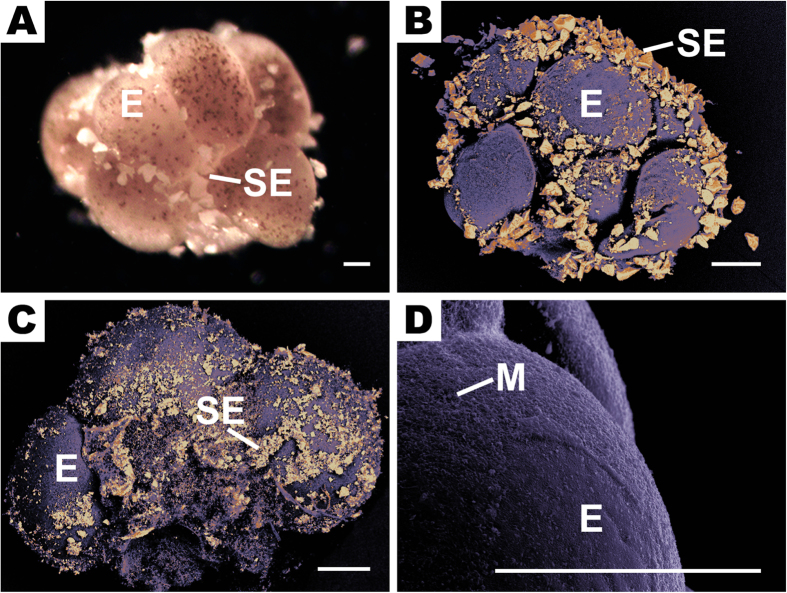
Microscopy images of coral egg-sperm bundles after failed ascent through elevated concentrations of suspended sediments, revealing considerable attachment of sediment grains to the bundles. Attached sediments hampered the ascent of these otherwise positively buoyant bundles. (**A**) Optical microscopy image showing sediment grains attached to a *M. digitata* egg-sperm bundle. (**B–D**) Colored backscatter scanning electron microscopy micrographs, showing sediment grains in yellow and biotic matter in purple. Shown are coral bundles of (**B**) *A. nasuta* and (**C**) *M. digitata.* Panel (**D**) shows the sticky mucous membrane, which thickens where the oocytes contact each other. All scale bars = 200 μm. E, egg; SE, sediment; M, mucous membrane.

Imaging of individual bundles revealed strong sediment attachment (Fig. 1). Light microscopy demonstrated preferential accumulation of sediment grains on the mucous coating between oocytes (Fig. 1A). Scanning electron microscopy yielded high-contrast images showing bundles covered in a tangle of sperm, sediment and mucus (Fig. 1B,C), clearly distinguishing sediments (yellow) from biotic matter (purple). The mucous coating was thickest at the junctures between oocytes (Fig. 1D).

**Figure 2:**
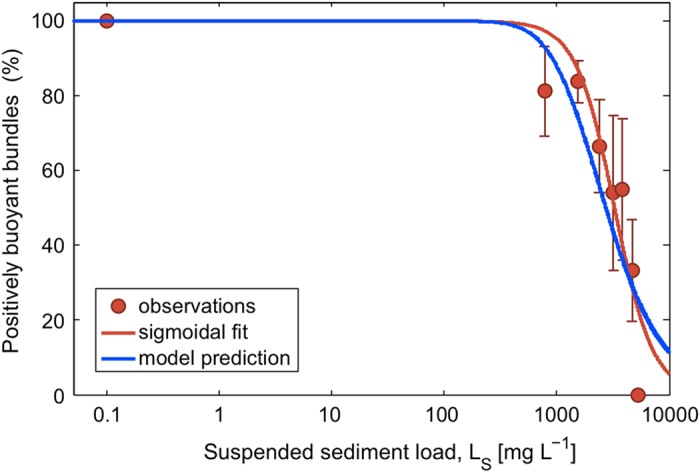
The fraction of positively buoyant egg-sperm bundles decreases with increasing sediment load. Red circles denote laboratory observations of the fraction of *M. digitata* bundles successfully rising through a 77 cm tall water column, for different values of the total sediment load, *L*_S_. Error bars are standard errors (s.e.m.) computed over 4 runs containing replicates of at least 3 bundles each. The red curve is a sigmoidal fit to the observations, given by *y* = 100 {1 + 10^[2.537 (log_10_*L*_S_ − 3.514)]} ^−1^. Also shown is the prediction from a physically-based Monte Carlo model (blue line) that quantifies the ballasting of rising bundles by sediment grains encountered by direct interception, for the same conditions as in the experiments (bundle radius *r*_B_ = 489.6 ± 54.6 μm (mean ± s.d.); bundle rising speed *v*_B_ = 6.35 ± 1.37 mm s^−1^; sediment grain radius *r*_s_ = 25.6 ± 8.8 μm; sediment density ρ_S_ = 2500 kg m^−3^).

The fraction of ascending bundles decreased nonlinearly with increasing SS load (Fig. 2), with 50% of bundles failing to ascend at a SS load of EC_50,A_ = 3262 mg L^−1^ (95% c.i.: 2523–4218 mg L^−1^). Taken together with the visual evidence of sediment attachment (Fig. 1A–C), these results support the proposed mechanism of sediment ballasting.

Ascent failure predictions from the model are in excellent agreement with our laboratory observations over the full range of SS loads tested (Fig. 2). For the 77 cm tall water column used in experiments, the model’s prediction of EC_50,A_ = 2768 mg L^−1^ is within 20% of the experimental value of 3262 mg L^−1^ (95% c.i.: 2523 – 4218 mg L^−1^), and the predicted and observed dependence of ascent failure on SS load were statistically indistinguishable (*F* = 1.853, *p* = 0.1655). This agreement further supports our hypothesis that the ballasting from sediments intercepted by a rising bundle can terminate its ascent, and validates the use of the model to predict sediment ballasting under natural conditions.

**Figure 3:**
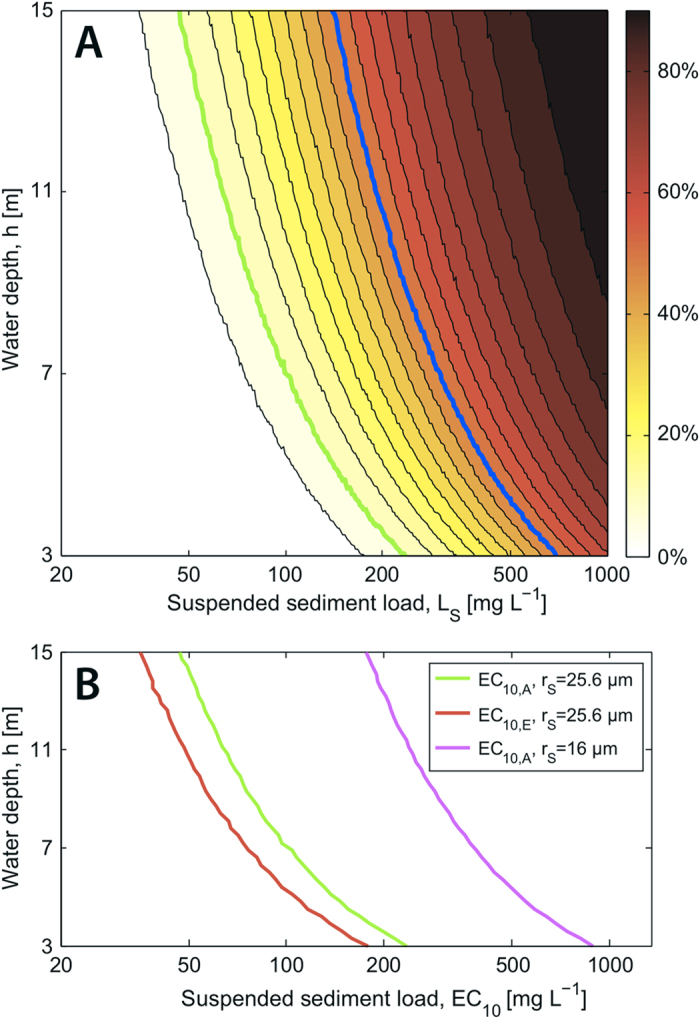
Reduction in the number of coral egg-sperm bundles reaching the water surface due to sediment ballasting, predicted by a model of sediment ballasting as a function of water depth, *h*, and sediment load, *L*_S_. (**A**) Percent reduction. The green and blue contours denote the SS loads that reduce successful ascents by 10% (EC_10,A_) and 50% (EC_50,A_), respectively. (**B**) EC_10_ values for ascent failure (EC_10,A_; green and purple lines, referring to two different sediment grain radii *r*_S_) and encounter failure (EC_10,E_, red line), as a function of water depth, *h*. Note that EC_10,E_ < EC_10,A_ because the decrease in egg-sperm encounters is quadratic in the decrease in ascents. For both panels, model parameter values are as in Fig. 2, except for sediment grain radii *r*_S_ in panel B, indicated in the legend.

When applied to typical water depths at which corals live, the model shows that considerable reductions in the fraction of ascending bundles occur even at modest sediment loads (Fig. 3A). For example, the model predicts EC_10,A_ = 236 mg L^−1^ for *h* = 3 m and EC_10,A_ = 47 mg L^−1^ for *h* = 15 m (Table 1). The ascent failure increases with SS load and with water depth, in both cases due to the larger number of sediment grains encountered by a bundle before reaching the surface (Fig. 3A, Table 1).

Because bundles carry both sperm and eggs, ascent failure has a quadratic effect on egg-sperm encounter rates at the surface, with direct repercussions on fertilization rates. The effect is quadratic because encounter rates are proportional to the product of egg and sperm concentrations at the surface and each bundle that fails to ascend negatively impacts both. This is evident in the SS loads causing a certain (*e.g*., 10%) reduction in encounters (EC_10,E_) being lower than the SS loads causing the same reduction in ascents (EC_10,A_): for example, EC_10,E_ = 179 mg L^−1^ < EC_10,A_ = 236 mg L^−1^ for *h* = 3 m and EC_10,E_ = 35 mg L^−1^ < EC_10,A_ = 47 mg L^−1^ for *h* = 15 m (Fig. 3B, Table 1).

Bundles ascending through suspensions of smaller sediments are less sensitive to ascent failure (Fig. 3B), because the grain radius *r*_S_ strongly impacts bundle-grain encounters (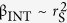, Eq. 1). For instance, sediments with *r*_S_ = 16 ± 5 μm (mean ± s.d.) result in EC_10,A_ = 891 mg L^−1^ for *h* = 3 m and EC_10,A_ = 177 mg L^−1^ for *h* = 15 m, ~4-fold greater than corresponding values for *r*_S_ = 25.6 ± 8.8 μm grains (Fig. 3B).

**Figure 4:**
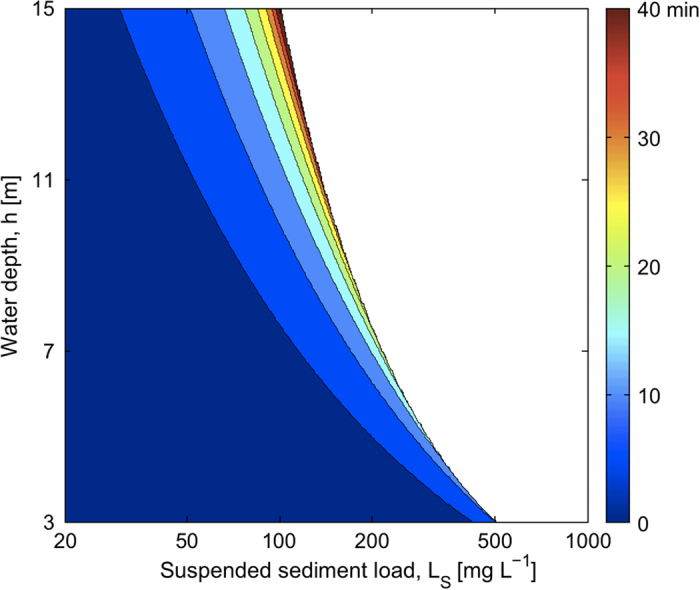
Time delay in the ascent of those coral egg-sperm bundles that successfully reach the surface, as a function of water depth, *h*, and suspended sediment load, *L*_S_. Model parameter values as in Fig. 2.

Sediment ballasting can be detrimental even when ascent succeeds, because decreased buoyancy reduces rising speed and thus increases time to surface. A quantification of the ascent time obtained by dynamically tracking the bundle’s buoyancy yields delays of <10 min for corals at 3 m depth, but up to 40 min for corals at 15 m depth at high SS loads (Fig. 4). Bundles spawned from deeper colonies are thus at greatest risk of missing adequate gamete concentrations for fertilization at the surface.

The ascent failure of coral egg-sperm bundles is a previously unrecognised mechanism threatening coral recruitment if spawning overlaps with conditions of elevated SS loads, such as those associated with dredging activities or natural resuspension events. The ballasting of coral gametes during ascent adds to the cumulative risk that sediments pose on early life history stages including reduced fertilisation, larval development and settlement^7^,^11^, and could contribute to recruitment failure on nearby reefs. Our model predicts that bundles released from deeper corals have the greatest chance of sinking because of accumulation of sediment grains and that medium-coarse silt sediments (~25 ± 10 μm radius) result in a strong ballasting effect. Additionally, our work shows that finer silt-sized sediments (~16 ± 5 μm radius), which remain in suspension longer, can also sink bundles at elevated SS loads (~180 mg L^−¹^) at deeper water sites. Importantly, in all cases, ascent failure not only results in less reproductive material reaching the water surface, it also translates to a quadratic decrease in egg-sperm encounters, which will directly impact fertilisation success (Fig. 3B). As recruitment is necessary to sustain populations and facilitate recovery from disturbances, recruitment failure may have a long-lasting negative legacy on a given reef^10^.

Sediment ballasting is not strongly dependent on the bundle’s rising speed and may thus be relatively insensitive to variations in rising speed among bundles from different species. This is due to the dual effect of a faster rising speed, which enhances encounters with grains per unit time (Eq. 1) but reduces the ascent time (*h*/*v*_B_, Eq. 2). Faster rising speeds are in fact predicted to somewhat reduce the number of sediment grains encountered (Eq. 2). More consequential for ascent success are the bundle’s density at release, *ρ*_B_, and size, *r*_B_, with larger and more buoyant bundles withstanding greater sediment ballasts (Eq. 3). A smaller bundle density is intuitively beneficial. A larger size is beneficial because it increases buoyancy while, counter intuitively, not increasing the encounter rate with sediment grains (*r*_B_ does not appear in Eqs 1 and 2; Methods). Coral species with larger egg-sperm bundles are thus predicted to fare better under heavy SS load conditions.

Sediment ballasting can be aggravated by additional encounter mechanisms beyond direct interception. Whereas Brownian motion is typically negligible (see Methods), relative fluid motion in highly turbulent environments (turbulent kinetic energy >10^−6^ W kg^−1^) could contribute several percent to direct interception (Methods), rendering actual encounter rates somewhat higher than predicted here. On the other hand, not every encounter between a bundle and a sediment grain will result in attachment. Based on the high stickiness of the mucus enveloping egg-sperm bundles^12^,^13^, we have assumed 100% capture efficiency of encountered sediment grains; the strong agreement between experimental data and modelling outcomes supports this approach for *M. digitata*. Any reduction in the capture efficiency due to lower adhesion between sediments and bundle or to limited mucous coverage of the bundle, will result in a proportional decrease in the encounter rate and thus an increase in EC_10_ values.

Even when sediment loads are insufficient to sink bundles, they cause ascent delays that may reduce reproductive success. Spawning synchrony is crucial for achieving an adequate sperm concentration at the water surface^14^, and mixing significantly reduces fertilisation probabilities as little as 1 h after spawning^15^. With such a narrow fertilization window, the ~40 min ascent delay predicted here for bundles from the deepest corals (Fig. 4) would be ecologically significant. Subtle but consistent differences in the spawning times of closely-related species is also thought to be a mechanism for reproductive isolation, preventing or reducing hybridization^16^. Loss of punctuality and blurring of the fertilization window could reduce the efficacy of that important prezygotic isolation barrier.

The laboratory observations and mathematical simulations presented here indicate that ascent failure is probable for bundles from all but shallow-water colonies if spawning occurs in proximity to turbidity-generating processes. This is apparent when comparing the EC_10_ and EC_50_ values computed here (Table 1) with reported SS loads from the environment. The latter include values of 50–840 mg L^−^¹ measured within a few kilometres from dredging and disposal operations^17^,^18^,^19^ and values >100 mg L^−^¹ caused by natural resuspension events in shallow lagoonal areas^1^,^20^. Furthermore, river discharge loads over inshore reefs can exceed 1200 mg L^−1  ^21, indicating that sediment ballasting could be particularly relevant to coral spawning in fringing habitats.

To predict the risks posed by turbidity-generating activities such as dredging to the spawning of corals, and possibly of other species relying on buoyant eggs for fertilization^22^,^23^,^24^, we propose that sediment ballasting be integrated in ocean circulation and sediment transport models. This will couple sediment resuspension, advection and settling with sediment interception by rising bundles, so that the SS loads at any given site can be used to produce local risk maps for coral fertilization. These findings describe a novel threat to coral reefs, yet they also present a practical approach for improving management of sediment-generating activities during sensitive coral spawning events.

## Methods

To test the effect of different SS loads on the ascent of egg-sperm bundles, we quantified the fraction of bundles from the digitate coral *Montipora digitata* successfully ascending through a 77 cm tall acrylic column containing different loads of carbonate sediments (radius *r*_S_ = 25.6 ± 8.8 μm, mean ± s.d.) suspended in 0.4 μm-filtered seawater at 28 °C. After sediment-laden water was added to the column, 3–5 freshly collected and intact egg-sperm bundles were transferred to the top of the column and the column was inverted, allowing bundles to rise. The number of bundles ascending was assessed after 164 s (the time by which 95% of bundles surfaced in sediment-free water). Four replicate runs, for a total of ≥12 bundles, were performed for each SS load. Bundles from *M. digitata* and *Acropora nasuta* were also exposed to elevated SS loads and examined by light and scanning electron microscopy to determine attachment of sediment grains (SI Methods).

To further test the mechanistic basis of sediment ballasting and predict its consequences in natural conditions, we developed a mathematical model based on encounter rate theory for differentially settling particles: the bundle of radius *r*_B_ rising with speed *v*_B_ and the sediment grains of radius *r*_S_ sinking with speed *v*_S_. The dominant encounter mechanism is ‘direct interception’ (SI Methods), which occurs when the center of a sediment grain comes within one grain radius of the bundle. The encounter kernel β_INT_ , given by^25^


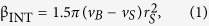


represents the equivalent volume of seawater from which all sediment grains are ‘captured’ by a rising bundle per unit time. This yielded the number of sediment grains encountered by the bundle during its ascent time *h*/*v*_B_ (where *h* is the water depth),


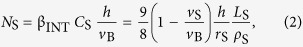


where *C*_S_ = *L*_S_/*M* is the concentration of sediment grains, *L*_S_ the SS load, *M* = (4/3)πρ_S_*r*_S_^3^ the mass of one grain, and ρ_S_ = 2500 kg/m^3^ the sediment density. These grains ballast the bundle, whose density, *ρ*, becomes


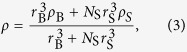


where *ρ*_B_ is the sediment-free bundle density (SI Methods). When 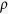 exceeds the seawater density, the bundle sinks. For the sizes and densities of sediments and bundles used in this study (SI Methods), approximately 50 sediment grains are sufficient to sink a bundle.

For each scenario, 50,000 bundle ascents were modelled using a Monte Carlo approach to take into account variability in key parameters. Parameter values and their distributions were obtained from our experimental observations and literature (SI Methods). This allowed us to quantify the fraction of failed ascents and the critical SS loads EC_10,A_ and EC_50,A_ (the SS loads causing 10% and 50% ascent failure).

## Additional Information

**How to cite this article**: Ricardo, G. F. *et al.* That sinking feeling: Suspended sediments can prevent the ascent of coral egg bundles. *Sci. Rep.* **6**, 21567; doi: 10.1038/srep21567 (2016).

## Supplementary Material

Supplementary Information

## Figures and Tables

**Table 1:** Concentrations of suspended solids predicted to cause a 10% (EC_10_) and a 50% (EC_50_) reduction in bundle ascent (subscript A) and gamete encounter (subscript E) for coral bundles of *M. digitata*.

Water depth [m]	Ascent failure		Encounter failure	
	EC_10,A_ [mg L^−1^]	EC_50,A_ [mg L^−1^]	EC_10,E_ [mg L^−1^]	EC_50,E_ [mg L^−1^]
15	47	141	35	87
10	71	211	53	131
5	141	422	106	262
3	236	699	179	436
0.77 (Model)	911	2726	690	1676
0.77 (Expt)	—	3262	—	—

Model parameters as in Fig. 2.
