# Supplementary material for: That sinking feeling: Suspended sediments can prevent the ascent of coral egg bundles
Source: Sci Rep. 2016 Feb 22;6:21567. doi: 10.1038/srep21567 (PMC4761919; doi:10.1038/srep21567)
Supplement: Supplementary Information [file srep21567-s1.pdf]

Supplementary Information

That sinking feeling:
Suspended sediments can prevent the ascent of coral egg bundles

Gerard F. Ricardo, Ross J. Jones, Andrew P. Negri and Roman Stocker*

* To whom correspondence should be addressed. E-mail: romanstocker@ethz.ch

This Supplementary Information includes:

SI Materials and Methods

Supporting Table S1

SI Materials and Methods

Model formulation and choice of parameters. An egg-sperm bundle begins its journey following the release from a polyp, and rises to the water surface aided by buoyant lipid-rich eggs [1]. We consider the rise of an individual egg-sperm bundle from the seabed to the water surface, through a water column of depth h having a sediment load L_S. The bundle is assumed to be spherical with radius r_B and to rise with speed v_B. We compute the total number of individual sediment grains, N_S, encountered by the bundle before it reaches the surface, using results from encounter rate theory. As the bundle rises, it displaces the water and sediment grains directly ahead of itself, and only 'captures' the grains that come within one grain radius of the bundle's surface. This mode of encounter is called 'direct interception' [2] and is the dominant encounter mechanism for this process (see below for a quantification of other encounter mechanisms, whose inclusion generally further increases sediment ballasting). Based on the stickiness of the mucopolysaccharides that coat eggs in corals and other species [3,4], we assume that each grain that encounters a bundle sticks to it. The bundle becomes heavier with each grain captured and when its density exceeds that of seawater, ascent fails.

Model runs were based on parameter values obtained from experimental observations and literature. In particular, we used a bundle radius r_B = 489.6 ± 54.6 μm and a bundle rising speed v_B = 6.35 ± 1.37 mm s^{-1} (in line with experimental values, see Table S1). The initial bundle density (*i.e.*, the density of the 'clean' bundle, without any sediments attached), ρ_B, is computed from the bundle size and rising speed using Stokes' law. The sediment grains are assumed to be uniformly distributed in the water column and spherical, with radius r_S = 25.6 ± 8.8 μm (matching the distribution used in the experiment) and density ρ_S = 2500

kg/m^3 (appropriate for calcareous material). This results in a sediment grain sinking speed $v_S = -(2/9)(g/\nu)[(\rho_S-\rho_F)/\rho_F]r_S^2$, where g is the acceleration of gravity, ρ_F = 1022 kg m^{-3} is the seawater density and ν = 8.79 x 10^{-7} m^2 s^{-1} is the seawater kinematic viscosity (ρ_F and ν were computed for a temperature of 28°C and a salinity of 35 PSU (practical salinity units)).

For direct interception, encounter rate theory prescribes the encounter kernel to be

$$\beta_{\mathrm{INT}} = 1.5\pi(v_B - v_S)r_S^2, \quad \text{(S1)}$$

which represents the equivalent volume of seawater from which all sediment grains are 'captured' by a rising bundle per unit time. β_{INT} depends on the difference in velocity between the bundle and the sediment grains: the contribution of rising by the bundle and sinking by the sediment grains is thus additive. Furthermore, β_{INT} is (counter intuitively) independent of the bundle size, r_B, due to the fact that a larger bundle displaces more water out of its path as it rises [2]. This formulation of β_{INT} is valid when sediment grains are much smaller than the bundle, $r_S << r_B$, as is the case here, and for bundles rising at small Reynolds numbers [2]. Although the bundle's Reynolds number is $r_B v_B/\nu$ ~3, and thus this criterion is not strictly satisfied, we do not expect this departure to significantly affect the computed encounter rates.

To determine the number of sediment grains N_S captured by a bundle over its journey from the seafloor to the water surface, we multiply the encounter rate kernel β_{INT} (volume/time) by the concentration of sediment grains C_S (number of grains/volume) and by the time required for the ascent (time), which is the water depth h divided by the bundle's rising speed v_B, *i.e.*

$$N_{\mathrm{S}} = \beta_{\mathrm{INT}}\, C_{\mathrm{S}}\, \frac{h}{v_{\mathrm{B}}} = \frac{9}{8}\left(1 - \frac{v_{\mathrm{S}}}{v_{\mathrm{B}}}\right)\frac{h}{r_{\mathrm{S}}}\frac{L_{\mathrm{S}}}{\rho_{\mathrm{S}}}, \quad \text{(S2)}$$

where we used $C_S = L_S/M$ and $M = (4/3)\pi\rho_S r_S^3$ is the mass of a single sediment grain. From the number and density of individual grains, we can compute the density of the sediment-ballasted bundle (the total mass of the bundle-sediment aggregate divided by its total volume) and, thus, its density difference with respect to seawater:

$$\Delta\rho = \frac{r_{\mathrm{B}}^3\rho_{\mathrm{B}} + N_{\mathrm{S}} r_{\mathrm{S}}^3\rho_S}{r_{\mathrm{B}}^3 + N_{\mathrm{S}} r_{\mathrm{S}}^3} - \rho_F. \quad \text{(S3)}$$

When $\Delta\rho > 0$, the bundle is negatively buoyant and fails to rise to the surface. We note that, in general, we would need to dynamically update the rising speed v_B of the bundle, which diminishes as the bundle becomes more ballasted. However, computations in representative cases showed negligible differences in ascent failure, which in fact increases

slightly when one updates v_B dynamically in the simulations, because the slower ascent allows more time for sediment capture.

To obtain the fraction of bundles that fail to reach the surface, we implemented the model above using a Monte Carlo approach. For each condition (*e.g.*, each water depth *h* and sediment load L_S), we simulated 50,000 bundles and for each bundle we chose the bundle radius, bundle ascent speed, and sediment grain size from a Gaussian distribution modelled on the distribution recorded in the experiments: for the bundle radius r_B the distribution was 489.6 ± 54.6 µm (mean ± s.d.); for the bundle ascent speed v_B the distribution was 6.35 ± 1.37 mm s^{-1}; for the sediment grain radius r_S the distribution was 25.6 ± 8.8 µm. For selected simulations, we varied the sediment grain size distribution to predict the effect of medium silt, having radius r_S = 16 ± 5 µm. The number of bundles for which the model predicted a failed ascent, out of the 50,000 simulated, was taken as the fraction of failed ascents.

Other encounter mechanisms. Our analysis of sediment ballasting has focused on direct interception as the mechanism by which bundles encounter sediment grains. Here we briefly consider the contribution of other encounter mechanisms that in general act simultaneously to direct interception. We show that under most circumstances they are negligible, justifying a focus on direct interception alone in quantifying sediment ballasting.

We first show that Brownian motion is a negligible encounter mechanism compared to direct interception for all but the smallest sediment grains. The encounter rate kernel for Brownian motion is $\beta_{BROW} = 4\pi(D_B+D_S)(r_B+r_S)$, where D_B and D_S are the Brownian diffusion coefficients of a bundle and a sediment grain, respectively [2]. Because $D_B << D_S$ and $r_B >> r_S$, we write $\beta_{BROW} \approx 4\pi D_S r_B$. Furthermore, from the Stokes-Einstein relation for the Brownian diffusion coefficient, we find $D_S = 0.22\text{x}10^{-13}$ m^2 s^{-1} for a sediment grain radius r_S = 10 µm. The relative importance of encounters by Brownian motion and by direct interception is then $\beta_{BROW} / \beta_{INT} = (4\pi D_S r_B) / [1.5\pi(v_B-v_S)r_S^2] < 10^{-4}$, signifying that encounters by Brownian motion are negligible.

We next show that the contribution of turbulence to the encounter rate is typically very small compared to direct interception, but can enhance encounters under conditions of very strong turbulence, further contributing to sediment ballasting. Turbulence favours encounters not by transporting particles per se, but by differentially transporting particles that are in close proximity to one another. The encounter rate kernel for turbulent encounters between a bundle and sediment grains is $\beta_{TURB} = 9.75\gamma r_B r_S^2 (1+ r_S/r_B)^3 / (1+ 2r_S/r_B)^2 \approx 9.75\gamma r_B r_S^2$ [2], which is valid when the sediment grain is much smaller than the bundle ($r_S << r_B$), as is the case here. Here, γ is the Kolmogorov shear rate. The relative

importance of encounters by turbulent fluid motion and by direct interception is then β_{TURB} / β_{INT} = $(9.75\gamma r_B) / [1.5\pi(v_B - v_S)]$. Under weak to moderate turbulence ($\gamma < 0.1$ s^{-1}), β_{TURB} / β_{INT} < 0.02. Under moderately strong turbulence ($\gamma = 1$ s^{-1}), β_{TURB} / β_{INT} ~ 0.16, indicating that strong turbulence can increase encounter rates by several percentage points compared to the case of direct interception alone, and may thus further exacerbate sediment ballasting.

From bundle ascent failure to egg-sperm encounter failure. Bundles carry both sperm and eggs to the ocean surface. The encounter process between the two gametes, once they have reached the ocean surface, results from a complex combination of motility, chemotaxis and fluid flow. Although this makes the encounter rate kernel difficult to quantify, the encounter rate will nevertheless be proportional to C_{SPERM} x C_{EGG}, the product of the concentrations of the two gametes. Therefore, the relative decrease in the gametes' encounter rate due to a decrease in the fraction of bundles reaching the surface can be readily calculated. For example, if the bundles reaching the surface decrease by a fraction q (*e.g.*, 20%), this implies a reduction by a fraction q (20%, in this example) in both C_{SPERM} and C_{EGG}, reducing the gametes' encounter rate to a fraction $(1-q)^2$ (= 64%, in this example) of their original value. The effect of failed ascent on failed gamete encounter is thus nonlinear (in the example, a 20% reduction in ascent causes a 36% reduction in egg-sperm encounter rate).

Ascent delay. Even if bundles reach the surface successfully, sediment-ballasted bundles do so with delay, due to the reduction in rising speed. To compute this delay, we modified the mathematical model so as to track the buoyancy and thus the rising speed of the bundle dynamically, by computing the density of the sediment-laden bundle at each instant of the upward trajectory to account for the sediments encountered up to that point. This computation was repeated for different water depths and sediment loads. Unlike the computation of the percentage of bundles reaching the surface, the computation of the time delay was deterministic.

Sediment preparation. Offshore carbonate reef sediments were collected from Davies Reef, Great Barrier Reef, Queensland (18.830°S, 147.631°E). Sediments were milled and screened to a median particle size of 26 µm and further processed to select for larger silt size-classes by wet sieving through a 32 and 63 µm aperture metal sieves. The sediment was then dried and combined with 0.4 µm filtered seawater (FSW), 35 PSU at 28°C to obtain SS loads of 0 – 7000 mg L^{-1}. The sediment-laden water was added to the column immediately before the experiment. Three 100 mL replicate samples were taken of each sediment concentration drained from the column and measured gravimetrically using 0.4 µm polycarbonate filters dried overnight in a 60°C oven to calculate final SS values.

Coral and gamete collection. Colonies of *Montipora digitata* (Dana) >15 cm in size were collected from 1–3 m depth from Hazard Bay, Orpheus Island, Great Barrier Reef (18.636°S, 146.496°E) in March 2014. Gravid colonies were transported to the Orpheus Island Research Station and placed in flow-through tanks at 29°C. Colonies were isolated shortly before spawning and the egg-sperm bundles pipetted from the surface and immediately used in experiments. Colonies of *A. nasuta* (Dana) >20 cm in size were collected from 3-5 m depth in October 2014 from Magnetic Island (19.157°S, 146.861°E), an island located in the central inshore Great Barrier Reef region. Corals were collected under the Great Barrier Reef Marine Park Authority Permit G12/35236.1.

Ascent experiment. The ascent success of *M. digitata* bundles was measured in 77 cm tall, 7 cm diameter acrylic columns on the nights of 18–22 March 2014. To determine typical bundle ascent rates and sizes, columns were filled with 0.4 µm filtered seawater (FSW) and 3-5 intact egg-sperm bundles were transferred to the top of the column. The column was carefully sealed to prevent the entry of any air bubbles and then inverted. Bundle ascent to the surface was timed and bundles were then immediately transferred to a plastic petri dish and their size measured with a stereo microscope (Olympus SZ61). To determine the ballasting due to sediment loads, bundles were similarly added the column at each treatment level and allowed sufficient time to reach the top of the column (164 s, a time calculated from when 95% of the bundles had surfaced in sediment-free seawater) and the proportion of buoyant bundles assessed. This was repeated over four replicate runs until a minimum of 12 bundles had passed through the column at each treatment level. The average ascent success for each treatment was plotted against the measured SS load (Fig. 2). A four-parameter logistic equation, constrained between 0 and 100% (see caption of Fig. 2), was used to fit the data and quantify the SS load that reduced ascent success by 50%, $EC_{50,A}$ (Table 1). Minor flocculation of sediments was sometimes observed in the water column, giving rise to variability between replicates, but use of a high number of treatments over the entire SS range enabled $EC_{10,A}$ and $EC_{50,A}$ values to be calculated with adequate confidence. To compare the experimental and theoretical data, the model was run using the same level of treatments and replication as the experiment, fit to a four-parameter logistic equation constrained between 0 and 100%, and compared by applying the *F* test in GraphPad Prism (v5, San Diego, USA).

Ballasted bundles were very fragile and could usually not be removed intact from the column. Therefore, to observe if and where sediment grains can adhere to bundles, bundles were mixed in a 400 mL vessel at a high SS load (7000 mg L^{-1}). The vessel was then inverted twice, and ballasted bundles were pipetted from the bottom and observed under the stereo microscope or prepared for scanning electron microscopy by fixing in 1.25% glutaraldehyde and 0.5% paraformaldehyde in FSW. Samples were dehydrated in a microwave using a

graded ethanol series (70%, 90% × 2, 100%, 100% (anhydrous)) for 40 s at 250 W, and then critical point dried (Polaron KE3000, Quorum Technologies) in liquid CO_2 at 31.1°C and 1072 PSI. The samples were then mounted on carbon tape on aluminium stubs and coated with 3 nm platinum and imaged using a field emission scanning electron microscope (Zeiss 55-VP).

Table S1. Radius of egg-sperm bundles and their measured ascent rates for four coral species in sediment-free seawater.

Species	Bundle radius, µm mean ± s.d. (*n*)	Bundle ascent rate, mm s^{-1} mean ± s.d. (*n*)
Acropora millepora	729 ± 38 (6)	5.8 ± 1.4 (12)
Acropora nasuta	641 ± 139 (11)	4.6 ±1.3 (12)
Acropora tenuis	620 ± 30 (10)	4.3 ± 1.1 (13)
Montipora digitata	489 ± 55 (12)	6.4 ± 1.4 (12)

SI References

1 Arai, I. *et al.* Lipid composition of positively buoyant eggs of reef building corals. *Coral Reefs* **12**, 71-75, (1993).

2 Kiørboe, T. *A mechanistic approach to plankton ecology*. (Princeton Univ. Press, 2008).

3 Okubo, N. & Motokawa, T. Embryogenesis in the reef-building coral *Acropora* spp. *Zool. Sci.* **24**, 1169-1177, (2007).

4 Riehl, R. & Patzner, R. A. Minireview: the modes of egg attachment in teleost fishes. *Ital. J. Zool.* **65**, 415-420, (1998).
